# Monitoramento da Congestão Sistêmica na Insuficiência Cardíaca: A Avaliação Clínica é Suficiente?

**DOI:** 10.36660/abc.20240354

**Published:** 2024-07-15

**Authors:** Silvia Moreira Ayub-Ferreira, Danielle Louvet Guazzelli

**Affiliations:** 1 Hospital das Clínicas da Faculdade de Medicina da Universidade de São Paulo Instituto do Coração São Paulo SP Brasil Instituto do Coração do Hospital das Clínicas da Faculdade de Medicina da Universidade de São Paulo, São Paulo, SP – Brasil

**Keywords:** Insuficiência Cardíaca, Ultrassonografia, Hospitalização, Readmissão do Paciente

O tratamento da insuficiência cardíaca aguda descompensada (ICAD) continua a ser um desafio significativo em cardiologia, particularmente na prevenção de readmissões hospitalares.^1^ Neste contexto, os sinais e sintomas relacionados com a congestão estão entre as causas mais comuns de hospitalização por insuficiência cardíaca e subsequentes readmissões.^2^

Nesta edição dos Arquivos Brasileiros de Cardiologia^3^ os autores apresentam um estudo de coorte que incorpora o uso do escore VExUS para avaliar a congestão venosa residual antes da alta hospitalar (Score VExUS na alta hospitalar como preditor de readmissão em paciente com insuficiência cardíaca agudamente descompensada: Um estudo de coorte). Este estudo destaca a complexidade do manejo de pacientes com insuficiência cardíaca e sugere uma ferramenta potencialmente útil para melhorar os resultados clínicos.

A congestão venosa é um dos principais preditores de readmissão em pacientes com ICAD, e a capacidade de avaliá-la com precisão e eficácia pode permitir intervenções mais direcionadas e potencialmente reduzir as taxas de readmissão hospitalar.^4^ O uso do escore VExUS como medida quantitativa do congestionamento é particularmente promissor, pois torna a avaliação desta condição mais sensível. Esse escore avalia a dilatação da veia cava inferior e utiliza Doppler pulsátil das veias hepática, porta e intrarrenal para fornecer um quadro abrangente do estado de congestão do paciente.^5^

O estudo^3^ adotou uma metodologia de coorte prospectiva, envolvendo pacientes adultos com fração de ejeção do ventrículo esquerdo igual ou inferior a 40%, sintomas de classe funcional II a IV da New York Heart Association e evidência clínica de congestão venosa com necessidade de diuréticos intravenosos. A avaliação do escore VExUS foi realizada imediatamente antes da alta hospitalar (a equipe atendente foi responsável por comunicar a intenção de alta). O endpoint primário considerado foi um composto de readmissão ou visitas de emergência devido a ICAD dentro de 90 dias após a alta.

O estudo^3^ constatou que, dos 49 pacientes analisados, 34,7% apresentavam escore VExUS 2 ou 3 na alta. Esses pacientes apresentaram proporção significativamente maior de readmissões ou visitas de emergência em comparação com aqueles com pontuação VExUS 0 (35,3% versus 9%, p=0,044). Estes dados sugerem que uma pontuação VExUS elevada na alta está associada a um risco aumentado de eventos adversos pós-alta, indicando uma área potencial de intervenção para otimizar a gestão do congestionamento antes da alta.

Estudos anteriores, como os realizados por Torres-Arrese et al., não encontraram utilidade prognóstica para o escore VExUS na alta hospitalar.^6^ No entanto, este estudo indica que o escore pode identificar pacientes com maior risco, sugerindo uma diferença provavelmente devida a características do paciente, como maior gravidade da doença neste caso.

Apesar dos achados promissores, o estudo^3^ apresentou limitações, inclusive o tamanho da amostra, o que pode afetar a generalização dos resultados. No entanto, seu estudo é um dos primeiros a investigar o escore VExUS como ferramenta prognóstica na alta de pacientes com ICFEr e sugere que uma avaliação detalhada da congestão venosa pode ser crucial para melhorar o manejo da ICAD.

Este estudo^3^ expande a compreensão da complexidade do manejo da ICAD e destaca a necessidade de mais pesquisas para validar a pontuação VExUS como um alvo para a terapia de descongestão e para melhorar os resultados dos pacientes. A integração das medidas da VCI e dos parâmetros Doppler no escore VExUS pode aumentar a precisão da avaliação da congestão sistêmica residual, um passo potencialmente benéfico na abordagem da insuficiência cardíaca descompensada.

Concluindo, este estudo^3^ piloto ilustra a importância de avaliações precisas da congestão venosa em pacientes com ICAD e sugere que o escore VExUS pode ser uma adição valiosa às ferramentas de avaliação clínica. A investigação futura deverá centrar-se não apenas na validação deste escore num espectro mais amplo de pacientes, mas também no desenvolvimento de estratégias de intervenção baseadas nas suas indicações para melhorar o prognóstico de pacientes com insuficiência cardíaca.
