# Supplementary material for: Monitoramento da Congestão Sistêmica na Insuficiência Cardíaca: A Avaliação Clínica é Suficiente?
Source: Arq Bras Cardiol. 2024 Jul 15;121(5):e20240354. [Article in Portuguese] doi: 10.36660/abc.20240354 (PMC11463341; doi:10.36660/abc.20240354)
Supplement: Translation

# Translation: Monitoring Systemic Congestion in Heart Failure: Is Clinical Evaluation Sufficient?

**DOI:** 10.36660/abc.20240354i

**Authors:** Silvia Moreira Ayub-Ferreira, Danielle Louvet Guazzelli

**Affiliations:** 1 São Paulo SP Brazil Instituto do Coração do Hospital das Clínicas da Faculdade de Medicina da Universidade de São Paulo, São Paulo, SP – Brazil

**Keywords:** Heart Failure, Ultrasonography, Hospitalization, Patient Readmission

The management of acute decompensated heart failure (ADHF) remains a significant challenge in cardiology, particularly in preventing hospital readmissions.^1^ In this context, signs and symptoms related to congestion are among the most common causes of hospitalization for heart failure and subsequent readmissions.^2^

In this edition of *Arquivos Brasileiros de Cardiologia*^3^ the authors present a cohort study that incorporates the use of the VExUS score to assess residual venous congestion before hospital discharge (VExUS Score at Hospital Discharge as a Predictor of Readmission in Patients with Acutely Decompensated Heart Failure: A Cohort Study). This study highlights the complexity of managing patients with heart failure and suggests a potentially useful tool to improve clinical outcomes.

Venous congestion is one of the main predictors of readmission in patients with ADHF, and the ability to accurately and effectively assess it may allow for more targeted interventions and potentially reduce hospital readmission rates.^4^ The use of the VExUS score as a quantitative measure of venous congestion is particularly promising, as it makes the assessment of this condition more sensitive. This score evaluates the dilation of the inferior vena cava and uses pulsed Doppler of the hepatic, portal, and intrarenal veins to provide a comprehensive picture of the patient's state of congestion.^5^

The study^3^ adopted a prospective cohort methodology, involving adult patients with a left ventricular ejection fraction of 40% or lower, New York Heart Association functional class II to IV symptoms, and clinical evidence of venous congestion requiring intravenous diuretics. The VExUS score assessment was performed immediately before hospital discharge (the attending team was responsible for communicating the intention to discharge). The primary endpoint considered was a composite of readmission or emergency visits due to ADHF within 90 days after discharge.

The study found that, of the 49 patients analyzed, 34.7% had a VExUS score of 2 or 3 at discharge. These patients showed a significantly higher proportion of readmissions or emergency visits compared to those with a VExUS score of 0 (35.3% versus 9%, p=0.044). These data suggest that a high VExUS score at discharge is associated with an increased risk of adverse events post-discharge, indicating a potential area of intervention to optimize congestion management before discharge.

Previous studies, such as those conducted by Torres-Arrese et al., did not find prognostic utility for the VExUS score at hospital discharge.^6^ However, this study indicates that the score can identify patients at higher risk, suggesting a difference probably due to patient characteristics, such as a greater severity of disease in this case.

Despite the promising findings, the study^3^ had limitations, including the sample size, which may affect the generalization of the results. However, his study is one of the first to investigate the VExUS score as a prognostic tool at discharge for patients with HFrEF and suggests that a detailed assessment of venous congestion may be crucial for improving the management of ADHF.

This study^3^ expands the understanding of the complexity of managing ADHF and highlights the need for more research to validate the VExUS score as a target for decongestion therapy and to improve patient outcomes. The integration of IVC measurements and Doppler parameters in the VExUS score may enhance the accuracy of assessing residual systemic congestion, a potentially beneficial step in the approach of decompensated heart failure.

In conclusion, this pilot study^3^ illustrates the importance of accurate assessments of venous congestion in patients with ADHF and suggests that the VExUS score may be a valuable addition to clinical assessment tools. Future research should focus not only on validating this score across a broader spectrum of patients but also on developing intervention strategies based on its indications to improve the prognosis of patients with heart failure.
